# Key determinants to school breakfast program implementation in Philadelphia public schools: Implications for the role of SNAP-Ed

**DOI:** 10.3389/fpubh.2022.987171

**Published:** 2022-10-11

**Authors:** Elisabeth G. Fornaro, Erin McCrossan, Peter Hawes, Ebru Erdem, Gabriella Maria McLoughlin

**Affiliations:** ^1^Office of Research and Evaluation, School District of Philadelphia, Philadelphia, PA, United States; ^2^College of Public Health, Temple University, Philadelphia, PA, United States; ^3^Implementation Science Center for Cancer Control and Prevention Research Center, Brown School, Washington University in St. Louis, St. Louis, MO, United States

**Keywords:** school meals, breakfast, implementation science, policy, nutrition insecurity, qualitative

## Abstract

**Background:**

Policies addressing food insecurity are only effective if they are implemented successfully, serving those most at risk. Universal school meals provide a key intervention to schools that serve predominantly low-income families by providing free school breakfast and lunch to all. Unfortunately, low uptake of such provisions among students is concerning especially regarding school breakfast, warranting key implementation support for schools to ensure student nutrition needs are met. Thus, the purpose of this study was to evaluate the determinants of implementing two different school breakfast programs and pragmatic strategies for serving breakfast in ways that maximize student participation.

**Methods:**

A qualitative study was conducted between 2018 and 2020 within the School District of Philadelphia (SDP) comprising surveys, interviews, and observations to assess contextual determinants of two distinctive breakfast models: Breakfast in the Classroom (BIC) and Cafeteria after the Bell (CAB). Principals and lead kitchen staff completed surveys to assess determinants of breakfast model adoption. Principals, lead kitchen staff, classroom teachers, climate (i.e., social emotional wellbeing), and facilities staff subsequently participated in interviews to discuss implementation determinants (i.e., facilitators and challenges) and strategies for maximizing student participation. Observations provided rich data to triangulate interviews and survey data. Survey data were analyzed using frequency analysis, and observation and interview data were analyzed through thematic analysis. Presentation of themes was framed by the Consolidated Framework for Implementation Research.

**Results:**

Results highlighted several positive determinants to participation including addressing student and family needs, making data-informed decisions, and providing hot meals and fruit based on student tastes. Negative determinants to implementation comprised challenges to SNAP-Ed-funded policy changes to promote student breakfast participation, lack of communication between administration, and staff and turnover among food service staff. Strategies included modifying school entrance procedures and combining breakfast with other education-related tasks to minimize instructional time lost through breakfast after the bell schedules.

**Discussion:**

Data highlight the need to include implementation partner expertise when designing interventions for increasing reach and effectiveness of school meal programs. Future research that directly tests implementation strategies and key outcomes of reach/participation, among others, is critical to bridging the policy to practice gap in school nutrition programs.

## Introduction

Eating breakfast has a positive association with students' academic outcomes and attendance. Conversely, skipping breakfast is associated with decreased cognitive performance, such as alertness, attention, memory, and problem-solving (1–3). In an effort to promote student breakfast and lunch consumption at school, federal policies such as the Community Eligibility Provision (CEP) were enacted to provide free meals to schools and districts serving low-income student populations (4–6). Universal school breakfast programs can therefore mitigate food insecurity of families whose students are in the public school system; this is particularly true for urban schools and districts that serve students of racial/ethnic minority backgrounds and low-income families (7, 8). However, despite provision of CEP and school breakfast programming, low reach (i.e., participation rates) poses implementation challenges for schools and districts trying to meet the needs of their students (9–11). Thus, further research to elucidate the determinants of successful implementation is warranted to improve health benefits of food assistance policy.

Many models for breakfast service exist and are often chosen based on the needs of each individual school system (12). In addition to the traditional model of serving breakfast in the cafeteria before school, termed “cafeteria before the bell,” other options include serving breakfast on “grab-n-go carts;” serving “breakfast in the classroom” (BIC) after school starts; and serving breakfast in the “cafeteria after the bell” (CAB; see Table 1). In the last decade, several studies have been conducted to elucidate the impact of these various models on attendance, meal participation, nutritional outcomes, and even on student weight status (2, 13–18). All studies report that BIC or CAB are positively associated with improved attendance and participation, highlighting factors such as reduced stigma and accommodation of student/family needs in decision making as potential antecedents to such changes (2, 14, 18). However, research to date has mainly examined the impact of breakfast models on student outcomes, but not the factors which influence implementation of each model. Partnerships with Supplemental Nutrition Assistance Programs-Education (SNAP) funded programs are a potential key opportunity to facilitate breakfast implementation, yet evaluation into such partnership is lacking. Without such understanding of implementation determinants, our ability to develop implementation strategies to improve outcomes, such as reach and participation, is limited.

**Table 1:** Breakfast service models and definitions.

**Model**	**Definition**
Cafeteria before the bell	Breakfast is served in the cafeteria before school starts.
Grab-n-go cart	Breakfast is available on a cart in the hallway (or somewhere else in the building) before or after the bell.
Breakfast in the classroom (BIC)	Breakfast is delivered to classrooms for students to eat all together after school starts
Cafeteria after the bell (CAB)	Breakfast is served in the cafeteria after school starts, either to entire classrooms who come through the line together or to individual students who arrive late

The field of implementation science offers important insights for studying the implementation and utilization of evidence-based policies and programs (19, 20). Its application to the present study through application of the Consolidated Framework for Implementation Research (CFIR) (21, 22) provides a theoretical foundation to studying implementation determinants (i.e., facilitators and challenges) of school breakfast models, which is a key first step in development of implementation strategies for improving their impact on health outcomes. Specifically, the CFIR comprises five key domains and several constructs within such domains: (1) Innovation Characteristics (i.e., components of the breakfast model); (2) Outer Setting (i.e., factors outside the school context); (3) Inner Setting (i.e., within-school factors); (4) Characteristics of Individuals (i.e., school and staff); and (5) Implementation Process (i.e., getting implementation underway). Examining implementation determinants through these constructs will help to identify opportunities for support from leadership and researchers.

This study was conducted in the School District of Philadelphia (SDP) during the 2018–19 and 2019–20 school year to answer the following research questions: (1) What are the positive determinants to school breakfast model implementation and student participation in SDP schools? (2) What are the negative determinants to school breakfast model implementation and in what ways can they be mitigated to maximize student participation? and (3) What are pragmatic strategies that schools can implement to mitigate negative determinants and increase reach of breakfast programming?

## Methods

All research procedures were approved by the institution's Internal Review Board and all study participants (who were over 18 years of age at the time of the study) provided informed consent through signed documents. This article presents data from a 2-year study on school breakfast in SDP, including surveys completed by lead kitchen staff and principals, observations, and qualitative in-depth interviews at four school sites (Table 2). These different data sources were used together to understand the implementation processes, successes, and challenges of different breakfast delivery models adopted by SDP schools.

**Table 2:** School demographic information.

**School site**	**Breakfast model description**	**School type**	**Enrollment**	**Student demographics**	**Interviews**
1	“Cafeteria before the bell” (with a “Grab-n-go cart” in a multipurpose room before the bell due to space limitations in the cafeteria) <1 year with the current model (in year prior there was some BIC)	Elementary	640	27% English Language Learners 11% Students with IEPs 100% Economically Disadvantaged 0% American Indian 4% Asian 24% Black/African American 62% Hispanic 8% Multi-Racial <1% Pacific Islander 1% White	4
2	Mostly “cafeteria before the bell” with some “breakfast in the classroom” (special education classes eat BIC after the bell) Model in place for 5+ years	High	1,080	29% English Language Learners 33% Students with IEPs 100% Economically Disadvantaged 0% American Indian <1% Asian 31% Black/African American 64% Hispanic 2% Multi-Racial 0% Pacific Islander 1% White	2
3	Schoolwide “BIC” Model in place for 5+ years	Elementary	640	18% English Language Learners 7% Students with IEPs 100% Economically Disadvantaged <1% American Indian 4% Asian 21% Black/African American 69% Hispanic 2% Multi-Racial <1% Pacific Islander 4% White	2
4	“CAB” (with some “BIC” due to space constraints) Model in place for <1 year (in year prior served BIC)	Elementary	680	25% English Language Learners 7% Students with IEPs 100% Economically Disadvantaged <1% American Indian <1% Asian 18% Black/African American 77% Hispanic 3% Multi-Racial <1% Pacific Islander 2% White	2

### Implementation context: Philadelphia public schools

In large urban school districts, such as the SDP, every student has the option of eating free breakfast at school due to CEP which allows all schools/districts serving students with over 40% identified as low-income to provide free breakfast and lunch (4, 5). However, during the 2018–19 school year (before COVID-19), breakfast participation across the district averaged 42%. Due to its positive effects on attendance, cognition, and academic outcomes (1–3, 13, 23), in Fall 2017 the SDP set a goal of serving breakfast to 70% of students in attendance each day. This goal is important considering that the food insecurity rate for SDP student households was estimated to be 19.1% during the 2019–2020 school year (24). This rate was substantially higher than the rate estimated for the city as a whole (14.4%) as well as the rate for the state of Pennsylvania (10.2%) (25, 26). The high rate of food insecurity among SDP student households represents an unmet need within the district, and an opportunity to innovate breakfast meal service. SDP partnered with Eat Right Philly (ERP), the district's nutrition and wellness program. This organization is a federally funded program through USDA SNAP-Ed and works with SDP's Division of Food Services to support schools in increasing breakfast participation. We were particularly interested in discerning school's experiences with ERP and how the role of SNAP-Ed agencies could be strengthened in breakfast program implementation.

### Lead kitchen staff surveys

In spring 2019, SDP lead kitchen staff completed surveys to provide their experiences implementing school breakfast models. Lead kitchen staff manage the cafeteria and are the primary staff members responsible for implementing the school's chosen breakfast model. Grounded in the CFIR (22), these surveys focused on lead kitchen staff experiences with implementing various breakfast models at their schools. Example closed questions included: “Which of the following outside groups have promoted school breakfast participation at your school in the last year?” (outer setting) and “How important were the following factors in your school's decision to have BIC?” (innovation characteristics and inner setting). Options for outside involvement included the ERP, the city's SNAP-Ed provider. Additionally, there were 15 open-ended questions that invited respondents to explain more about implementation barriers and facilitators of specific breakfast models (BIC, Cafeteria Before the Bell, CAB); if lead kitchen staff would be interested in trying a model in the future; and if no, why not. The research team sent the survey to the 242 lead kitchen workers managing SDP cafeterias. A total of 145 lead kitchen staff took the survey, for a response rate of 60%.

### Principal surveys

In 2020, the research team sent surveys to SDP principals to understand the successes and challenges to implementing different breakfast delivery methods adopted by SDP schools to distinguish why implementation of BIC stopped. Prior research indicated that schools that offered BIC to all students had higher breakfast participation rates than schools that used other models (17). However, the implementation challenges associated with this model are unknown and perspectives of key implementers are therefore needed to identify key implementation determinants of adopting BIC and CAB. A total of 60 surveys were sent through email to principals at SDP schools. First, surveys were sent through email to a random selection of principals at 56 SDP schools. In addition, surveys were sent to the principals of the four schools where observations and interviews were taking place (described below). After an initial email and two reminder emails, principals at 38 schools responded to the survey for a response rate of ~63%. Six respondents (15.7%) did not complete the survey, but their responses to questions they did complete are included in the analysis. Most principal survey respondents had worked at their school “5–9 years” (31%).

### School observations and school staff interviews

During the 2019–20 school year, the research team conducted a total of 14 observations and 10 interviews at four SDP schools to understand the successes and challenges of different breakfast models. Given that the prevalence of food insecurity is one reason why it is vital to maximize breakfast participation rates, we first limited the sample (all SDP non-charter schools) to schools where there might be a greater need for augmented food security. To select schools where food security is a bigger concern, we used two criteria: (1) the school's Identified Student Percentage and (2) parent and principal responses to the 2017–18 District Wide Survey (DWS). School Identified Student Percentage data from 2018–19 determined the rate of students qualifying as economically disadvantaged determined according to their participation in specific benefit programs. Not all students or families participate in benefit programs for which they are eligible, which may result in an underestimate. We limited the sample to schools with an Identified Student Percentage rate of more than 75% of students qualifying as economically disadvantaged. To compare breakfast models, we purposefully selected two sites that implemented breakfast before the bell, one that implemented BIC, and one that implemented CAB. Information on each school type, enrollment, and breakfast service model is shown in Table 3. The School District had adopted CEP which allows schools and districts with an Identified Student Percentage above 40% to provide breakfast and lunch free of charge to students (4–6).

**Table 3:** Description of breakfast model by school.

**School site**	**School type**	**Enrollment**	**Breakfast service practices**
1	Elementary	640	• “Cafeteria before the bell” (with a “Grab-n-go cart” in a multipurpose room before the bell due to space limitations in the cafeteria) • Served 30 min before school starts and ended at the start of school • Less than 1 year with the current model (in year prior there was some breakfast in the classroom)
2	High	1,080	Mostly “cafeteria before the bell” with some “breakfast in the classroom” (special education classes eat breakfast in the classroom after the bell) • Served before school starts • Cafeteria stays open ~10 min past the start of school to serve late students • Model in place for more than 5 years
3	Elementary	640	Schoolwide “Breakfast in the Classroom” • Packaged in crates picked up by students from the kitchen at the start of school • Served after school starts school for ~30 min • Model in place for more than 5 years
4	Elementary	680	“Cafeteria After the Bell” (with some “Breakfast in the Classroom” due to space constraints) • Breakfast timing staggered by grade: ° 1st and 2nd ate breakfast in the cafeteria after school starts (two 1st grade classes ate breakfast in a second cafeteria space because they did not physically fit in the main cafeteria) ° 3rd and 4th went to their classrooms at the start of school and then came back to the cafeteria to eat when 1st and 2nd grade finished ° Kindergarten ate breakfast in a second cafeteria space after school starts • Model in place for <1 year (in year prior served breakfast in the classroom)

We also limited the sample to schools where 20% or more of parent/guardians who responded to the 2017–18 DWS answered “yes” to the question, “In the past 30 days, have you worried about having enough food for you or your family?” We chose this marker because the city-wide food insecurity rate was ~20% (25). Across SDP, 13% of parents and guardians who responded to the 2018–19 District-Wide Survey answered “yes” to the question “In the past 30 days, have you worried about having enough food for you or your family?” (27). The DWS is administered each spring to students, teachers, principals, and parents and guardians. The survey asks respondents about how they experience and perceive their schools. In 2018–19, 22% of SDP parents and guardians responded to the DWS (27). In addition, we limited the sample to schools with an enrollment of over 500, given that smaller schools would not have the same logistical issues as larger schools when serving breakfast. We then selected typical cases representing a combination of different breakfast models (Cafeteria before the bell, Grab-n-go cart, BIC, and CAB).

Formal, semi-structured interviews (28) were conducted at the four schools using interview protocols designed specifically for school administrators, teachers, kitchen staff, or school facilities staff. Interviews were conducted by two members of the research team either in person or over the phone. Interviews were recorded and transcribed, lasting ~20–45 min. Interviews aimed to gain insights on the specific factors within and outside the school setting that were influential to adoption and implementation of a particular breakfast service model. Questions included: “Who makes decisions about breakfast at your school and how?” “How is breakfast delivered and cleaned up at your school? What do you think about this delivery method?” and “What do you think the importance of breakfast is to student health, attendance, behavior, and/or academics? How do you think the delivery and/or clean-up model impacts these things?” School staff were also asked about their involvement with ERP and what this partnership looked like in their building, to elucidate how ERP might support implementation of school breakfast. Interview guides were developed in collaboration with the office of food service related to their experiences with breakfast implementation.

A total of 14 observations were conducted across the four schools. Observations were conducted between November 2019 and March 2020. Two research team members visited the school on each observation date in order to allow for observation of different breakfast service sites in the school (e.g., cafeteria, classroom, and multipurpose room). Trained research team members with extensive experience with Philadelphia schools and nutrition service took field notes during each observation (28, 29). Field notes documented breakfast procedures in real time and captured the context of breakfast at each school. The focus of field notes was to understand the ways in which schools approached specific breakfast models, as well as the challenges and benefits associated with specific breakfast models.

### Data analysis

Lead Kitchen Staff and principal surveys were completed using Qualtrics software. Data were analyzed descriptively to ascertain frequencies to understand the determinants of breakfast implementation factors. Interviews were recorded and transcribed verbatim. Interview transcripts and fieldnotes were analyzed thematically using Dedoose software (Los Angeles, CA) by three members of the research team and two research assistants. This approach was adopted to capture the rich contextual detail within each setting and to capture nuanced determinants of implementation that may not be present in the literature to date. To develop the codebook, all members of the research team read select interview transcripts and fieldnotes and noted any common concepts that emerged from the data(30). Several iterations of discussing and relating common concepts led to a final codebook of 13 data themes. Inter-coder reliability was established through three rounds of testing using the Dedoose training feature. All transcripts and fieldnotes were coded by one team member and checked by a second team member. The research team wrote informal memos about emerging categories and themes throughout the coding process as a form of an audit trail to enhance credibility of the findings (31). Themes were generated from the coded data and subsequently linked to CFIR constructs, following recommendations by Damschroder et al. (22, 32, 33), in order to frame our understanding of how findings related to implementation. This served as a critical step to achieve theoretical triangulation between the themes and the CFIR, increasing external validity (34).

## Results

Data from surveys of 38 SDP Principals and 145 lead kitchen staff provides an overview of the successes, challenges, and supports related to different school breakfast models. Data from principal surveys, observations, and school staff interviews are presented together. For each section, we provide each theme, developed through thematic analysis, grouped by specific components of the CFIR model to facilitate interpretation.

### Positive determinants to school breakfast implementation and student participation

Key facilitators were the high demand for supplemental nutrition through school breakfast, the potential of BIC and CAB to promote reach of breakfast programming and innovating how students enter school buildings to maximize participation. Further, the kinds of foods served were found as a significant factor in student acceptability and reach.

#### Inner setting: Implementation model driven by stakeholder needs

Salient to the Relative Priority construct within the Inner Setting domain (22) across all models, there was a perception that students relied on their schools to access breakfast. Data from interviews show that school staff (administrators, teachers, and other support staff) at all four school sites, representing various “before the bell” and “after the bell” breakfast models, perceived school breakfast as the main way students were eating in the morning. For example, a school staff member at school site 1, which served breakfast in the cafeteria before the bell, felt that their school community viewed school breakfast as the main way students eat in the morning: “I think our community as a whole, I think that's their breakfast. It's not optional. You go to school. You eat breakfast. That's where you eat your breakfast” (School Staff Member, Interview, School Site 1).

Data from interviews and observations at school sites show BIC or CAB reduced barriers to students accessing school breakfast, such as having to arrive at school early or stigma associated with eating breakfast at school. For example, an administrator at school site 3, which served BIC school-wide, explained that parents and guardians face financial and time barriers to serving students breakfast at home before school:

We're in a high poverty school. A lot of working parents, a lot of grandparents raising their kids. A lot of kids' parents are getting off shift work and then bringing their kids to school. A lot of homes can't actually afford adequate nutrition for their students. Therefore, a lot of times breakfast is skipped, or parents rely on breakfast as a way for their students to get food, because what they're getting at home is infrequent because they can't afford it, they don't have the time, it's not nutritious. At least when they come to school, they get that nutritious well-balanced breakfast (Administrator, Interview, and School site 3).

**Figure 1:**
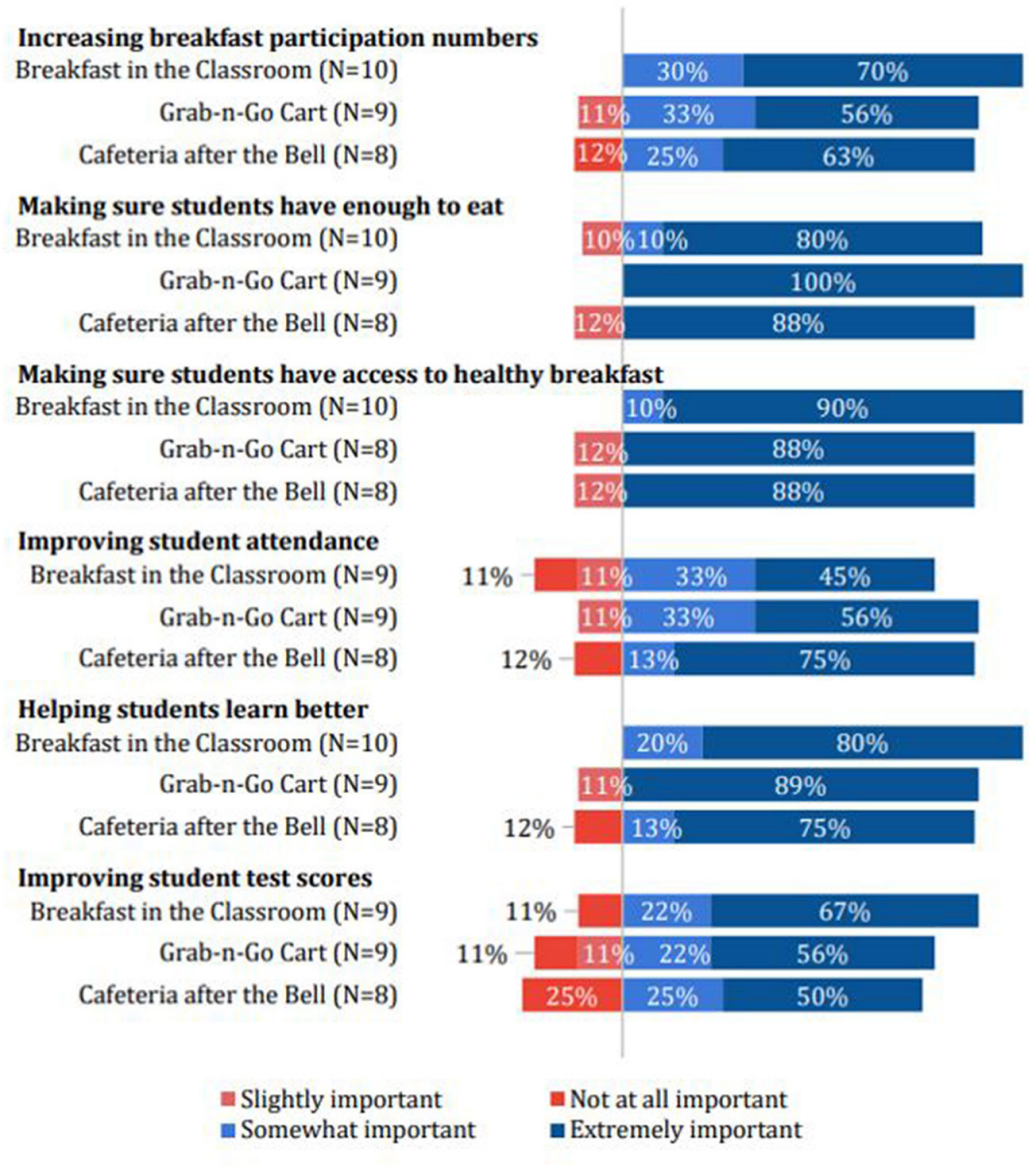
Relative advantage of school breakfast model choice.

This administrator found that serving BIC ensured students were well-fed because it removed barriers to breakfast participation, such as having to arrive at school early; serving CAB also removed this barrier. During observations of BIC and CAB, most students ate breakfast with their classes. Data from principal surveys showed that schools chose different breakfast models (BIC, “grab-n-go carts,” or CAB) to meet the same goals (Figure 1). When asked to identify which factors were most important for choosing their breakfast model, principals' responses were broadly similar across different breakfast models. The two most important factors overall were “making sure students have enough to eat” and “making sure students have access to healthy breakfast foods.” Considering only responses from schools with BIC and CAB, principals placed high importance on ensuring students have access to enough healthy breakfast foods. Additionally, there were some differences between BIC and CAB responses. Principals at schools with BIC placed slightly more importance on increasing participation numbers and helping students learn better. Principals at schools with CAB placed slightly more emphasis on attendance (Figure 1).

#### Implementation process: When “after the bell” breakfast models cannot be provided, having students enter the school through the cafeteria maximized participation

Pertinent to the Planning construct in the Implementation Process domain (22), in open-ended survey responses, principals emphasized the importance of students walking directly past breakfast options as they enter school. They referred to requiring students to enter through the cafeteria and/or the placement of a grab-n-go cart near the main entrance as important factors for student participation. School sites 1 and 2, which both served most of their students' breakfast in the cafeteria before the bell, had their students enter through the cafeteria.

School site 1 had third- and fourth-grade students enter through the cafeteria where breakfast was served before the bell. During observations, students entered, sat down at tables, and were called by table to go through the cafeteria line. Students were able to choose from cold (e.g., yogurts, cereals, and pastries) or hot (e.g., egg and sausage sandwich) breakfast items in addition to fruits, milks, and juices. Due to space constraints in the cafeteria, fifth-grade students entered through a multipurpose room, where breakfast was served on a grab-n-go cart before the bell. During observations, these students also entered, sat down at tables, and were called by table. However, students were limited to cold packaged items that could be delivered on a cart (cereals and packaged pastries) in addition to fruits, milks, and juices. At this school, students stayed in the cafeteria until their teachers picked them up. School site 2, a high school, had students stay in the cafeteria until they left independently for their first period class. During observations at school site 2, students entered the cafeteria and chose breakfast items, such as parfaits, juices, fruit, and pastries, from a cafeteria window, similar to a food court. Moreover, students who came to school after first period also entered through the cafeteria. They were required to stay in the cafeteria until the end of the first period to not disrupt class. Breakfast was still served, giving late students the option of eating, maximizing participation.

#### Implementation process: Providing students with hot meals and fresh fruit increased breakfast participation

Salient to the Reflecting and Evaluating construct in the Implementation Process domain (22), principals and school staff emphasized food quality, such as the ability to provide hot meals, as an important factor for student participation. For instance, one teacher observed that there are specific meals that maximized breakfast participation and other meals that students did not eat:

I think your breakfast participation would go up 2-fold if we served stuff that the kids would enjoy eating. That's just my opinion. Like I said, I don't know if anyone believes the same as me, but I know even just around my school, you see it. Some breakfasts the kids eat, some breakfasts they don't eat (Teacher, Interview, and School site 1).

School and cafeteria staff noted that the breakfast meals they observed as most popular are hot breakfasts, such as egg sandwiches, and felt they should be served more often. One principal responded to an open-ended survey question by writing: “students love the sausage muffins, but they are not served often” (Principal, Survey). Observations indicated that students liked when fresh fruit, such as oranges, were served with breakfast. During one observation of BIC, students cheered when the teacher looked in the breakfast crate and announced there were oranges. However, during other observations of BIC classes of ~20 were only given 5 or 6 oranges causing the majority of students to have to go without. Interviews and observations suggest that identifying and serving the most popular options more frequently, and ensuring a ratio of one item per student, would increase breakfast participation. Staff found breakfast foods that were lower in carbohydrates and sugars to be best for students, and that items high in sugar have a negative impact on student behavior.

### Negative determinants to implementation and student participation

Below we present some important challenges which must be mitigated to improve reach and participation of school breakfast programming. These relate to issues of communication among schools and SNAP-Ed providers regarding breakfast promotion, divergence in priority among school administrators and nutrition leadership, and turnover among staff.

#### Outer setting: Divergent perspectives on the role of ERP in breakfast participation

Related to the External Policies and Incentives construct within the Outer Setting domain (22), survey results suggest that Eat Right Philly (ERP), which provides SDP schools with SNAP-Ed funded nutrition and wellness programming, was closely linked to implementation of BIC and CAB. Schools that implemented BIC and CAB were much more likely to report outside engagement in breakfast promotion from ERP. Observations at all four school sites, each with different breakfast models, and interviews with school staff show that ERP posted information about nutrition on bulletin boards and provided materials for parents/guardians to take home. However, interview participants were not aware of when or how ERP specifically promoted breakfast. During an interview, a teacher highlighted that breakfast promotion was made difficult for ERP because they were tasked with promoting meals when students do not like all of the options:

Kids, when they think of breakfast, in my opinion, they think of eggs and pancakes and waffles and cereal and oatmeal. They don't think of a piece of banana bread as breakfast… You can promote it all you want, but if it's something the kids don't like to eat, they're not going to eat it because someone tells them it's good for you (Teacher, Interview, and School site 1).

Nonetheless, school staff believed that ERP encouraging students to try new foods increased breakfast participation. One school support staff member felt that in providing nutrition education and food tastings, ERP is teaching students to try new foods, making it more likely for them to try breakfast items. “…even though some of the ingredients they're not familiar with, they get excited afterwards because they actually participate. They make it so they really want to try what they make” (School Staff Member, Interview, School Site 1). This school staff member felt that students want to try the foods they make with ERP, increasing their enthusiasm to try new foods.

#### Inner setting: Kitchen staff and school staff experienced breakfast models differently

**Figure 2:**
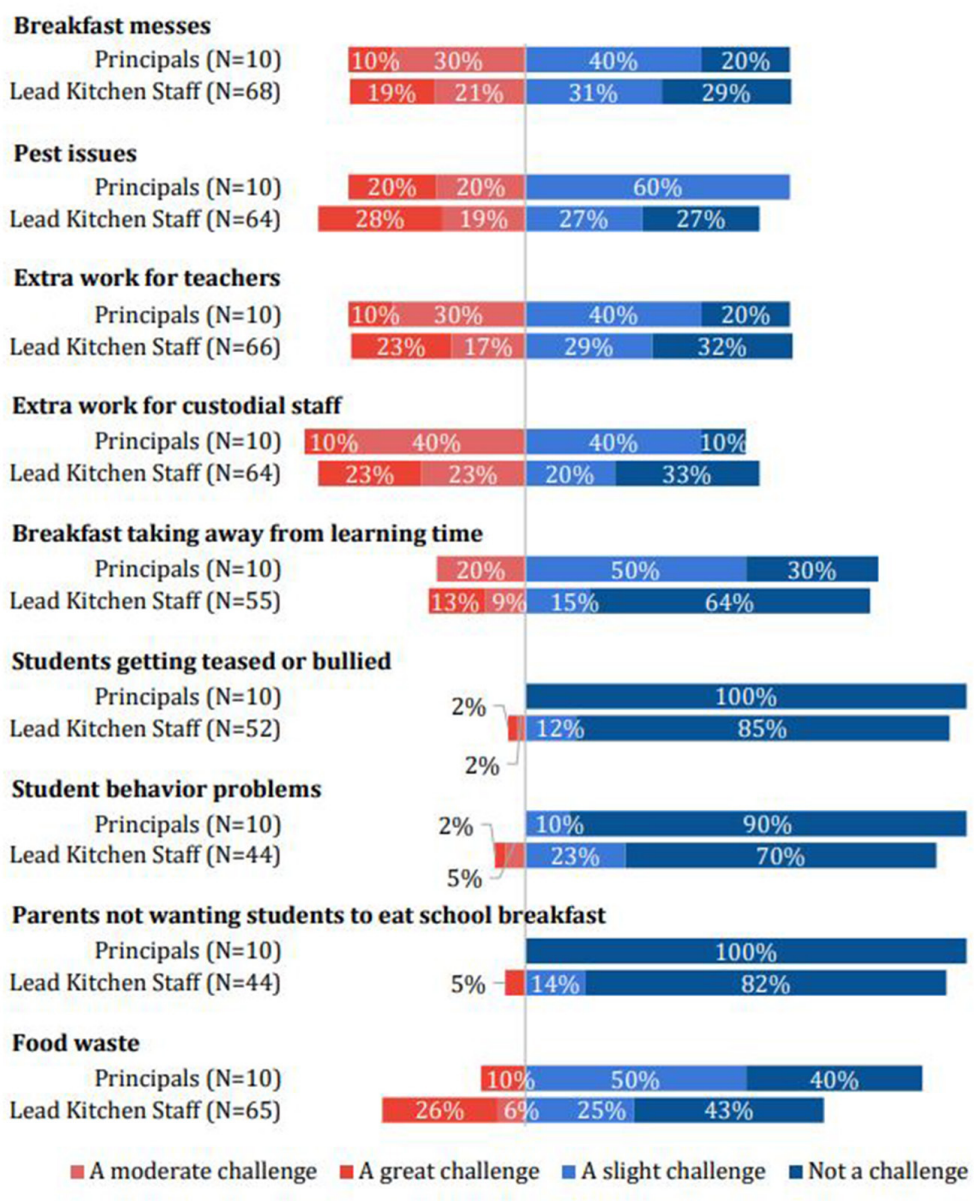
Perceived negative implementation determinants by respondent.

The Networks and Communications construct within the Inner Setting domain (22) highlights the difference among principal and lead kitchen staff survey responses, with some notable differences that may impact the breakfast model they implement. Principals gave the highest overall favorability rating to BIC while lead kitchen staff gave the highest rating to CAB. At surveyed schools that currently operate BIC, lead kitchen staff were more likely than principals to identify messes, pests (rodents and insects), extra work for teachers and staff as “great” challenges (Figure 2).

Other challenges, including food waste and student behavior, were also considered slightly more challenging by lead kitchen staff. Moreover, interviews indicate that communication between kitchen staff and school staff can be a challenge to successful breakfast implementation. For instance, an administrator at school site 3, which served BIC school-wide, expressed that the logistics of getting the breakfast crates to the classrooms, clean up once crates are returned, and recording breakfast participation requires communication about procedures:

We all see the value and the need to make sure that our students are well-fed, especially that starts with a really nutritious breakfast to start off the day. Any frustrations that come across usually come with procedural and lack of clarity (Principal, Interview, School Site 3).

#### Inner setting: Kitchen staffing challenges impeded consistency of breakfast delivery

Finally, within the Inner Setting domain are several constructs linked to Readiness for Implementation, which are illustrated by the lack of available resources such as staff and time for implementation (22). Inconsistent kitchen staffing impeded schools' ability to implement alternative breakfast models. At school site 2, which mostly served breakfast in the cafeteria before the bell, the lead kitchen staff member liked to serve fresh smoothies on a cart in the cafeteria, which was popular with students. However, when they did not have a complete kitchen staff, they were unable to do so: “unfortunately, I'm out of a cook and sometimes I'm out of a worker so that puts me behind the eight ball, so I have to stay in the kitchen” (Kitchen Staff Member, Interview, School Site 2). Kitchen staff turnover resulted in an inability to consistently serve breakfast in alternative ways found to be popular with students. Similarly, kitchen staff turnover also contributed to challenges with communication and coordination around procedures. As one administrator explained,

Sometimes I feel like my teachers aren't sure. Sometimes I feel like something's being said and then it changes based on rules and things like that. I think having [several] managers this year has... it's been a little bit stressful (Administrator, Interview, School Site 2).

Kitchen staff turnover can lead to changes in procedures, meaning that kitchen staff and school staff are no longer on the same page. School site 3, which served BIC school-wide, mitigated challenges to communication and coordinating by providing “refresher” trainings on breakfast procedures for school staff and holding meetings between kitchen staff and school staff.

### Potential strategies to mitigate logistical challenges in breakfast participation

Finally, we share some potential strategies that were observed/shared by SDP school staff which could be employed to address some logistical barriers to breakfast service. However, the needs of each school context and capacity of stakeholders must be considered.

#### Serving CAB to entire classrooms maximized participation while minimizing challenges

**Figure 3:**
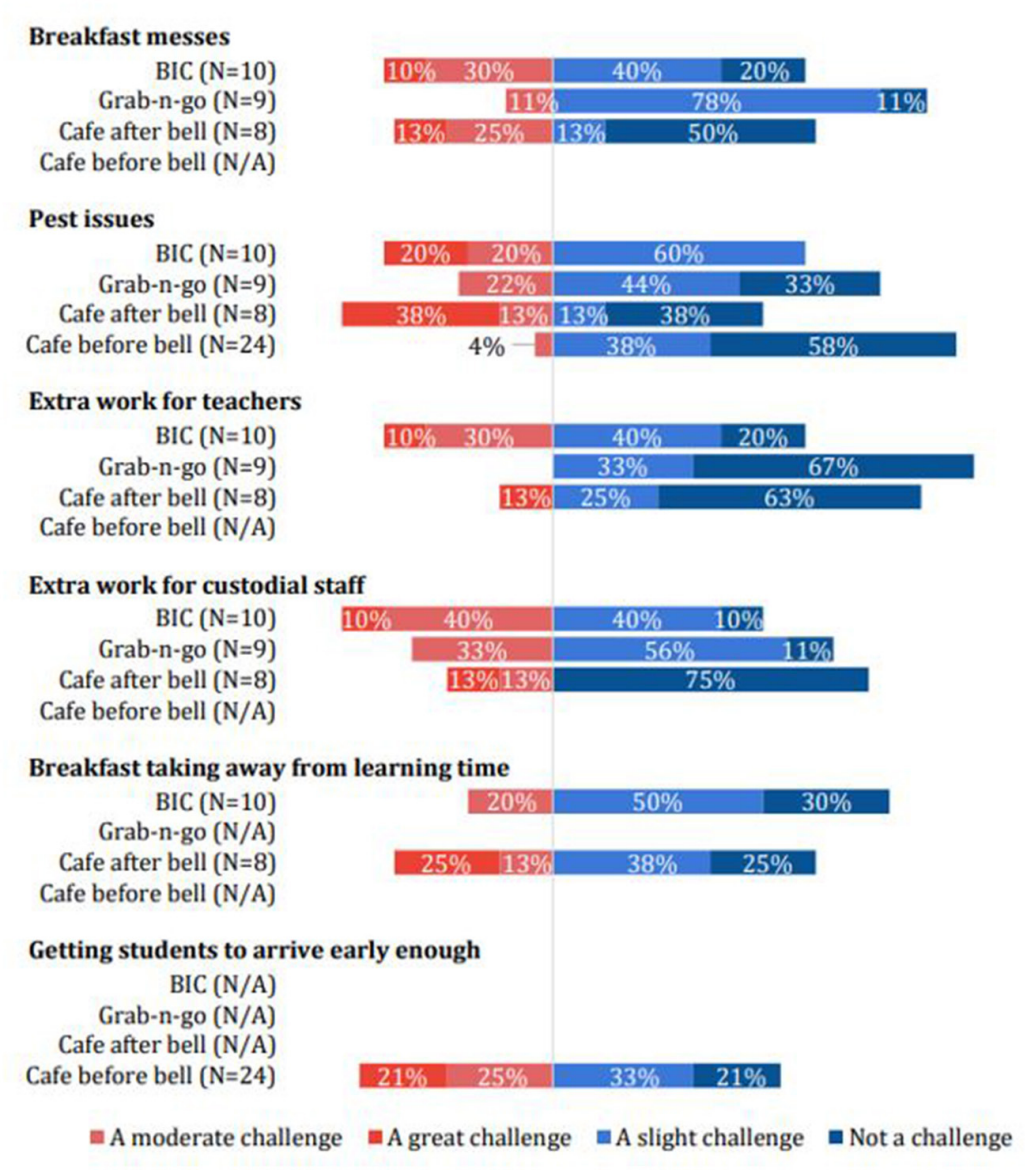
Principal-reported negative implementation determinants according to breakfast model.

Principal survey responses showed that most common challenges to breakfast implementation (e.g., messes, pests, or extra work for teachers and staff) were perceived to be less challenging by principals at schools that used the CAB model. However, getting students to come early was perceived as a significant challenge for schools with “cafeteria before the bell” (Figure 3). The BIC and CAB models removed the barrier of having to arrive at school early to be able to receive breakfast. Comparing the BIC and CAB models suggests that challenges were generally greater for BIC, on average (Figure 3). For some factors, such as messes and pests, both models had similar percentages of principals reporting “moderate” or “great” challenges. However, in these cases, CAB had much larger percentages reporting “not a challenge.” In other words, BIC was consistently rated as more challenging overall, while CAB was described as challenging only in some school contexts. BIC was closely associated with challenges related to messes, pests, and extra work for teachers and custodians. Schools implementing CAB reported greater challenges related to missed learning time. The results suggest that no single model is likely to be suitable in every school context. However, considering both survey and qualitative data, we find that the CAB model appears likely to address the primary concerns of school administrators, teachers, and support staff in many—but not all—school contexts.

Data from interviews with administrators and teachers show that classroom messes and pests were a challenge related to serving BIC. As discussed above, data from principal survey responses confirm this finding. For instance, an administrator at school site 4, which served breakfast in the CAB, and BIC to a few classes due to cafeteria space issues, cited mice as one reason for transitioning from BIC to the CAB model. A teacher at school site 3, which served BIC school wide, confirmed that cleaning up the classroom after breakfast was a challenge: “I like the fact that kids that may not be able to get to eat at home are able to eat, but the clean-up is a lot” (Teacher, Interview, School Site 3). The research team observed teachers and students sweeping up crumbs after BIC and one student cleaning up spilled milk. In addition, during observations of BIC, students had to leave the classroom to empty unused milk or juice into bathroom sinks. While messes are inevitable, serving CAB limits messes to one space.

In addition, staff indicated logistical barriers to serving hot breakfast meals, which were liked by students, in BIC and “grab-n-go cart” models. Data from interviews and observations show that serving hot breakfast using these models was too complicated. The principal at school site 3, which served BIC school wide, confirmed that serving hot BIC was “too hard.” Kitchen staff at school site 1, which used a grab-n-go cart explained that hot breakfast cannot be served on the grab-n-go cart because the temperature of the food would decrease as it was transported. Because BIC and “grab-n-go carts” made it very difficult to provide hot meals, CAB was the most feasible model for addressing this concern.

#### Integrating instructional time with CAB may mitigate time-related barriers

Data from principal survey responses showed that one concern with the CAB model was that students missed out on learning time. However, observation data provide potential strategies to address this challenge. At school site 4, which served breakfast in CAB to most classes (with two classes eating BIC due to space constraints), students ate breakfast in the CAB in two shifts. During the first shift, first- and second-grade classes ate breakfast in the cafeteria at 8:20 am. Two classes ate BIC due to space constraints. Kindergarten ate breakfast at the same time in a second cafeteria space. The research team observed K-2 students entering the cafeterias at 8:20 am, participating in morning announcements, and beginning to eat breakfast at ~8:30 am. Third and fourth graders went to their classrooms at 8:20 am and then came back down to the cafeteria during the second shift.

The research team observed both breakfast shifts which lasted ~20 min. All students in both shifts sat at one table together with their class and teacher, who facilitated breakfast service. The kitchen pre-prepared breakfast crates for each class and had them sitting on each table, which the teacher then passed out items and recorded participation for their class. Since students were sitting together with their classmates and teacher, eating breakfast in the CAB model provided time for announcements, learning activities, and community building. During the first breakfast shift, students listened to school announcements. Kindergarten students also participated in a literacy game. The research team observed classrooms eating together at a long cafeteria table. During an interview, a facilities worker at the school explained what they see in the cafeteria during “breakfast after the bell”:

A lot of kids, they sit around the table, they're all eating together. I couldn't really tell you how it impacts the little kids, I just see the expression on their faces, and they sit there, and they talk with their little friends, and they're having a good meal, so it's pretty good (School Staff, Interview, School Site 4).

At this school, the act of sitting together as a class with their teacher at one table in the cafeteria seemed to create a positive environment in a space conducive to eating and appeared to be a time for community building.

## Discussion

The purpose of this study was to identify the implementation determinants regarding two distinctive breakfast service models and potential mitigating strategies to enhance reach and participation within a large urban district and implications for potential future involvement of SNAP-Ed agencies. The use of the CFIR provided structure for the study design, analysis, and interpretation of findings which improved clarity and facilitated the use of data to drive future decisions on implementation support (22). Overall, school and food service staff perceived school breakfast as the main way students were eating in the morning and perceived BIC and CAB as a high priority for addressing food insecurity. Although “after the bell” models removed the barrier of students having to arrive at school early to eat, when they could not be provided, having students enter the building through the cafeteria maximized breakfast participation by explicitly inviting and encouraging students to eat. These findings reflect prior literature which highlights the positive impact of serving breakfast after the bell in promoting student participation, nutrition behaviors, and preventing absenteeism (13, 14, 35).

Within the inner setting domain, school staff also emphasized that food quality was an important factor in student breakfast participation with hot items, such as breakfast sandwiches, and fruit being particularly popular with students. It was not within the scope of our study to conduct interviews with students, but prior research indicates that perceptions of food quality and cultural relevance were key factors in participation in BIC initiatives (14) highlighting the importance of gaining student and parent input in breakfast programming and menu selection (36). In the current study, concerns were mainly from teachers/administrators regarding quality of food and a lack of high-protein options. These concerns are highly prevalent in other recent research with food service providers (37) and are linked to the reimbursement amount received for breakfast served which limit the procurement of high protein options (i.e., breakfast sandwiches) given the greater expense and preparation requirements for these foods. The United States Department of Agriculture (USDA) has recently increased reimbursement amounts for school breakfast and lunch (38) which may help to increase higher value options served at breakfast. Nonetheless, gaining student and parent input in decision making on menus may be a pragmatic strategy to increase participation.

To address another negative determinant within the Networks and Communications construct (22), due to the different perceptions among school staff and administration regarding breakfast model implementation, school and district leadership may also stress the importance of breakfast with school staff by sharing information on the positive association between breakfast and cognitive performance, academic outcomes, and attendance (7). Further, emphasizing the importance of school breakfast in addressing food insecurity by making sure school staff are informed of city-, district-, and school-level food insecurity rates could help improve adoption and implementation. Improved coordination between principals and lead kitchen staff could help identify challenges and the most appropriate breakfast model within each school context (18).

Finally, some key strategies emerged from schools that adopted BIC and CAB which provide advocacy support for schools who are deliberating adoption of these models, and for those who may be struggling to implement addressing barriers found in the Outer Setting domain of the CFIR (22). One of the key strategies to implementing BIC and CAB well was linked to collaborating with ERP. Principal survey data suggests that ERP involvement though delivering SNAP-Ed funded nutrition education in the classroom was closely linked to implementation of BIC and CAB breakfast models. This finding provides support for collaborating with SNAP-Ed agencies for promoting breakfast participation and reducing food insecurity in students and families (39–41). Recent findings show that more states are planning to use more policy, systems, and environmental (PSE) approaches in their SNAP-Ed programs to maximize the public health impact of this provision, highlighting opportunities for future research (39).

Furthermore, serving CAB to entire classrooms minimized challenges associated with the BIC model, such as messes and pests in the classroom, while still not requiring students to come to school early to eat. Combining educational practices with CAB reduces loss of instructional time and seems to improve participation in breakfast at school, and potentially mitigating challenges found within the Networks and Communications and Readiness for Implementation constructs (22). Organizations such as No Kid Hungry have issued guidance on how schools and districts can plan for and successfully implement breakfast after the bell which is inclusive of BIC and CAB (12), but such guidance does not include blending lessons with breakfast consumption. These data provide a potential pragmatic solution for CAB service; further research should be conducted to examine how participation and procurement could be impacted by this strategy.

### Implications for research and practice

Several implications arose from this evaluation. First, serving CAB to entire classrooms after school starts maximizes breakfast participation while minimizing challenges. To mitigate the amount of lost learning time, schools could consider serving breakfast in the CAB in two shifts, have students eat in the CAB together with their teacher and classmates, or provide each class a pre-prepared crate of breakfast meals to minimize the amount of learning time students miss. If schools cannot adopt either BIC or CAB due to logistical or contextual barriers, leadership may ask students to enter the building through the cafeteria to maximize participation so that every student must “opt out” of breakfast instead of opting in. To further incentivize participation, schools need to collect data from students regarding popular menu items and prioritize serving them. Considering the opinions of school-based staff and teachers who are with students during breakfast will also enhance implementation and overall school climate.

The present study identified several pertinent determinants which negatively impacted implementation of BIC, CAB, and other models. One key opportunity for partnership is to enhance communication between SNAP-Ed representatives and school food service providers, as one key goal of the SNAP-Ed program is to increase participation in school meals. This collaboration may drastically improve the implementation and uptake of school breakfast and empower school food service staff to address gaps with support of SNAP-Ed agencies such as ERP. Further investigation into feasibility is warranted but we urge researchers to consider their role as partnership builders in such process to increase the likelihood of success.

Finally, from a methodological standpoint, use of the CFIR facilitated understanding of these determinants and provided avenues for development of implementation strategies to bolster school/district capacity to implement breakfast models successfully (22). A critical next step for researchers who are partnering with school districts, especially urban districts, is to replicate our assessment of implementation determinants. Understanding the context-specific factors which influence adoption of evidence-based policies is essential to providing support. Findings from this study can inform data collection and analysis protocols and help researchers “narrow down” the specific factors to study. Subsequently, we highly recommend using rigorous implementation science methodologies to collaboratively develop and tailor implementation strategies (42, 43) to improve reach of breakfast programs and study their impact on school-level implementation outcomes and student behaviors. Such application will mark a necessary step in enhancing the public health impact of policies to address food insecurity.

## Data availability statement

The raw data supporting the conclusions of this article will be made available by the authors, without undue reservation.

## Ethics statement

The studies involving human participants were reviewed and approved by School District of Philadelphia Research Review Board. The patients/participants provided their written informed consent to participate in this study. Written informed consent was obtained from the individual(s) for the publication of any potentially identifiable images or data included in this article.

## Author contributions

EF and EM conceptualized the study and led data collection. PH and EE assisted with data collection and analysis. GM provided scientific consultation and led the writing of the manuscript. All authors provided substantial input on the manuscript. All authors contributed to the article and approved the submitted version.

## Conflict of interest

The authors declare that the research was conducted in the absence of any commercial or financial relationships that could be construed as a potential conflict of interest.

## Publisher's note

All claims expressed in this article are solely those of the authors and do not necessarily represent those of their affiliated organizations, or those of the publisher, the editors and the reviewers. Any product that may be evaluated in this article, or claim that may be made by its manufacturer, is not guaranteed or endorsed by the publisher.
